# Assessing the impact of social determinants of health on predictive models for potentially avoidable 30-day readmission or death

**DOI:** 10.1371/journal.pone.0235064

**Published:** 2020-06-25

**Authors:** Yongkang Zhang, Yiye Zhang, Evan Sholle, Sajjad Abedian, Marianne Sharko, Meghan Reading Turchioe, Yiyuan Wu, Jessica S. Ancker

**Affiliations:** 1 Department of Population Health Sciences, Weill Cornell Medical College, New York, NY, United States of America; 2 Information Technologies & Services Department, Weill Cornell Medicine, New York, NY, United States of America; Osakidetza Basque Health Service, SPAIN

## Abstract

**Objectives:**

Early hospital readmissions or deaths are key healthcare quality measures in pay-for-performance programs. Predictive models could identify patients at higher risk of readmission or death and target interventions. However, existing models usually do not incorporate social determinants of health (SDH) information, although this information is of great importance to address health disparities related to social risk factors. The objective of this study is to examine the impact of social determinants of health on predictive models for potentially avoidable 30-day readmission.

**Methods:**

We extracted electronic health record data for 19,941 hospital admissions between January 2015 and November 2017 at an academic medical center in New York City. We applied the Simplified HOSPITAL score model to predict potentially avoidable 30-day readmission or death and examined if incorporating individual- and community-level SDH could improve the prediction using cross-validation. We calculated the C-statistic for discrimination, Brier score for accuracy, and Hosmer–Lemeshow test for calibration for each model using logistic regression. Analysis was conducted for all patients and three subgroups that may be disproportionately affected by social risk factors, namely Medicaid patients, patients who are 65 or older, and obese patients.

**Results:**

The Simplified HOSPITAL score model achieved similar performance in our sample compared to previous studies. Adding SDH did not improve the prediction among all patients. However, adding individual- and community-level SDH at the US census tract level significantly improved the prediction for all three subgroups. Specifically, C-statistics improved from 0.70 to 0.73 for Medicaid patients, from 0.66 to 0.68 for patients 65 or older, and from 0.70 to 0.73 for obese patients.

**Conclusions:**

Patients from certain subgroups may be more likely to be affected by social risk factors. Incorporating SDH into predictive models may be helpful to identify these patients and reduce health disparities associated with vulnerable social conditions.

## Introduction

Early hospital readmissions are both common and costly [1]. For example, one in five patients enrolled in Medicare—a US public health insurance plan for people 65 or older or people with disability—is readmitted within 30 days after discharge, at a cost of over $26 billion per year [2]. Although some readmissions are unavoidable (e.g., regularly scheduled admissions for chemotherapy), a considerable proportion of readmissions are unnecessary and potentially preventable [3]. These readmissions are generally considered to indicate underlying issues with quality of care and can potentially be averted through appropriate interventions [1]. To improve the value of healthcare, federal, state, and commercial payers have included hospital readmission as one of the core quality measures in pay-for-performance programs. For example, under the Centers for Medicare and Medicaid Services (CMS) Hospital Readmission Reduction Program, hospitals face payment cuts if they have excess risk-standardized 30-day readmission rates relative to other hospitals [4].

Health disparities are of particular relevance to hospital readmissions because patients with low socioeconomic status (SES) and those living in disadvantaged neighborhoods are more likely to be readmitted [5–7]. Low SES and disadvantaged neighborhood conditions are among a number of potentially relevant social determinants of health (SDH) that are associated with increased risk of readmission, which include both individual-level social factors, such as patient education and income, and community-level factors, such as neighborhood unemployment and poverty rates [8, 9]. From a policy perspective, evidence related to the impact of SDH on hospital readmission has led to a controversy on whether readmission measures used by CMS to reimburse hospitals should adjust for socioeconomic factors in order to avoid penalizing hospitals for caring for disadvantaged patients, or whether doing so would inadvertently excuse the delivery of substandard care to disadvantaged populations [10–13]. To date, consensus remains elusive in this debate.

However, from the care delivery perspective, there is little doubt that accurately identifying patients who will be readmitted due to social risk factors can help reduce unnecessary healthcare utilization [14, 15]. Hospitals could target care management programs to socially disadvantaged patients to improve quality or partner with community organizations to address food, transportation, housing, or other social needs. Many health systems have been implementing both quality improvement strategies and programs to address social needs to reduce readmissions and improve quality of care, with promising results [15, 16].

One avenue that holds the potential to improve these strategies and programs is the inclusion of SDH in predictive models for 30-day readmission. Improved predictive modeling can be particularly helpful in low-resource settings, allowing community hospitals to effectively identify and target patients at high risk for potentially avoidable readmissions. A recent review identified 73 unique readmission prediction tools developed between 2011 and 2015 for a variety of patient populations and health conditions [17]. Health systems, such as accountable care organizations, have incorporated prediction algorithms in clinical routines for better care management [18, 19]. To date, only a few prediction models for hospital readmissions have incorporated individual-level SDH (e.g., age, gender, and race) or community-level SDH (e.g., median household income) predictors [20]. No study has examined if including both individual- or community-level SDH would improve the performance of prediction models. Without models that include these factors, hospitals lack not only valuable data about patient characteristics but also information about who to target in order to address social factors that may lead to adverse outcomes, including avoidable readmissions. Therefore, patients with disadvantaged social conditions may be underrepresented in care management programs, leading hospitals to miss an important opportunity to reduce unnecessary readmissions and improve care for these patients.

We hypothesized that the performance of a widely used hospital readmission risk prediction model would improve with the inclusion of individual-level and community-level SDH. We additionally hypothesized that the effects of adding SDH to predictive models would be greatest among the most vulnerable patient subgroups without resources to compensate for social risk factors. We therefore examined the performance of the model, and the impact of incorporating SDH, for three subgroups: patients receiving Medicaid (a US program granting subsidized care to low-income populations, often used as a proxy for low SES), obese patients, and patients 65 or older. Compared to other patients, these three groups of patients appeared likely to be disproportionately affected by vulnerable social conditions due to their low income, multiple chronic conditions, or disability.

## Materials and methods

### Study design, setting, sample, and data sources

This is a retrospective cohort study. Our goal was to update the Simplified HOSPITAL score, which predicts 30-day readmissions across disease conditions, with the inclusion of SDH data [21, 22]. To produce a cohort similar to those used to validate the Simplified HOSPITAL score, we identified adult patients discharged from medical services at an academic medical center in New York City between January 1, 2015 and November 30, 2017. Patients were included if they (1) had home addresses within the five boroughs of New York City, (2) were hospitalized for 24 hours or longer, (3) were not discharged to another medical center, (4) did not leave against medical advice, and (5) were alive at discharge.

Using existing institutional infrastructure for secondary use of electronic health record (EHR) data [23], we extracted data from the EHR at the academic medical center, including diagnosis, procedure, admission/discharge dates, discharge status, individual socioeconomic information, and 9-digit patient residential zip-codes. We collected SDH variables (listed below) at the US census tract level from various sources, including the US Census Bureau’s American Community Survey [24], Center for Disease Control and Prevention [25], United States Department of Agriculture, United States Environmental Protection Agency [26], the FACETS dataset [27], and New York City Open Data [28]. Census tract is a granular geographic unit typically containing between 1,200 and 8,000 residents [29].

The study was approved by Weill Cornell Medicine’s Institutional Review Board with a waiver of consent.

### Potentially avoidable hospital readmission or death

Our outcome is the potentially avoidable hospital readmission or death 30 days after an eligible hospital admission. We added death as part of our composite outcome, as early death after discharge also indicates adverse quality of care [30, 31]. To identify potentially avoidable hospital readmissions, we employed the 30-day all-cause unplanned hospital readmission algorithm from the Centers for Medicare and Medicaid Services (CMS), which is broadly used to identify index admissions and potentially avoidable readmissions in the U.S.[32]. Following this algorithm, we first excluded ineligible hospitalizations from index admissions, such as psychiatric admissions as these admissions are typically cared for in separate psychiatric or rehabilitation centers that are not comparable to short-term acute care hospitals. We also excluded transfers to another acute care hospital and patients who received palliative care during the hospitalization. For eligible index admissions, we identified all readmissions that occurred within 30 days after the discharge of index admissions. We then excluded planned readmissions, which are considered necessary and unavoidable. The CMS algorithm identifies planned readmissions based on three principles: (1) some types of care are always considered planned, such as transplant surgery, maintenance chemotherapy, and rehabilitation; (2) otherwise, a planned readmission is defined as a non-acute readmission for a scheduled procedure; and (3) readmissions for acute illness or for complications of care are not considered to be planned [32]. We incorporated two principal sources of data to identify patients who died within 30 days after discharge. In-hospital mortality was determined by internal ADT (admit/discharge/transfer) data. For deaths that took place outside of the hospital, we incorporated the Social Security Master Death File, matching on patient name and Social Security number.

### Prediction model for potentially avoidable 30-day hospital readmission or death

We applied the Simplified HOSPITAL score model to predict potentially avoidable 30-day hospital readmission or death [21, 22]. The original HOSPITAL score was developed and internally validated at a single U.S. academic hospital, then validated internationally at 9 hospitals across 4 countries [22]. The Simplified HOSPITAL score was developed and validated with similar prediction accuracy [33]. The predictors, all drawn from EHR data, include frequency of prior hospital admissions, urgency of admission, last available hemoglobin and sodium levels, discharge from an oncology division, and the index hospital length of stay (Table 1). We followed the methods established in the original HOSPITAL score algorithm and coded patients not tested for hemoglobin or sodium as normal for these two measures. The HOSPITAL score has achieved satisfying performance across diverse patient populations with a broadly varied range of reasons for initial hospitalization [21, 22, 33, 34].

**Table 1:** The Simplified HOSPITAL score model.

Predictors	Points if positive
Low **h**emoglobin level at discharge (<12 g/dL)	1
Discharge from an **o**ncology service	2
Low **s**odium level at discharge (<135 mmol/L)	1
**I**ndex admission **t**ype: urgent or emergent (non-elective)	1
Number of hospital **a**dmissions in the last 12 months	
0–1	0
2–5	2
>5	5
**L**ength of stay > = 5 days	2

### Social determinants of health

We extracted individual-level SDH from the EHR, including sex, race (defined according to US Office of Management and Budget standards, i.e., White, African American, Asian, American Indian/Alaska Nation, Native Hawaiian/Pacific Islander, other, and unknown), ethnicity (i.e., non-Hispanic, Hispanic, and unknown/declined/other), primary language (i.e., English vs. other), marital status (i.e., partnered vs. single), and insurance type (i.e., commercial, Medicare, Medicaid, dual-eligible for Medicare and Medicaid, and other public insurance). Prior studies have found that these variables are associated with increased risk of readmission [35–38]. For patients with missing race/ethnicity information in the EHR data, we coded their race/ethnicity as “unknown” as a separate category. Studies have indicated that patients with missing race/ethnicity have different characteristics as compared to patients with available race/ethnicity information [39, 40]. Coding missing patient race/ethnicity as an “unknown” category not only maintains a large sample size, but also is meaningful to indicate patients with different risks for adverse health outcomes as compared to patients with available race/ethnicity in the EHR data.

For community-level SDH, we first reviewed the relevant literature to identify SDH with a theoretical basis for potential association with readmission (S1 Table in S1 Appendix) [12, 41–44]. As community SDH variables are highly correlated, we assessed the collinearity among them by calculating Pearson’s correlation coefficients. Strongly correlated variables were excluded from the models. We also calculated variance inflation factors (VIF) to indicate the overall collinearity among predictors.

We selected variables that represent different domains of community social conditions, including socioeconomic status (i.e., median income, unemployment rate, % with high school or high school-equivalent diploma, % foreign born, % without insurance, and % dual-eligible); felony rate, walkability score, Gini income inequality coefficient, a composite score reflecting household composition and disability, and a composite score for minority status and language. S2 Table in the S1 Appendix indicates the sources of each SDH variables use in this study. A small number of patients (N = 45) with missing community-level SDH were excluded from the study.

### Statistical analyses

We first compared differences in demographics, comorbidities, and SDH between admissions with and without 30-day readmission or death. Demographic characteristics include age, age categories, and all the individual-level SDH (i.e., sex, race, ethnicity, primary language, marital status, and insurance type). Comorbidities include Charlson comorbidity score [45], HOSPITAL score, and body mass index (BMI). We also examine the admitting source of each admission (i.e., emergency department or other).

We first tested the performance of the Simplified HOSPITAL score using logistic regressions. We then ran three SDH-augmented models by adding: (1) all individual-level SDH drawn from the EHR; (2) Census tract-level neighborhood SDH; (3) All individual- and community-level SDH together. We ran these models using all patients, then performed subgroup analyses on Medicaid patients, patients 65 and older, and obese patients (BMI > 30). We used cross-validation to examine the predictive value of SDH. For each patient cohort (overall patient and three subgroups), we first randomly split the entire dataset into training set (75%) and testing set (25%). We conducted 3-fold cross-validation using training set. The whole training set was first partitioned into three near-equal parts. Three iterations of training and validation were then performed. Within each iteration, a model was trained on two parts, and then the fitted model was applied to the held-out part. The area under the receiver operating characteristic curve (AUC) was calculated on the held-out part. The model with the highest AUC on the held-out set was chosen as the final model. This model, with the same regression coefficients, was then applied to the remaining 25% of data to examine the performance of the model. We presented the regression results of models with both individual- and community-level SDH in the S3 Table in S1 Appendix.

For each model, we calculated the C-statistic for discrimination (> 0.7 indicates good discrimination), which refers to the ability to differentiate between admissions followed versus not followed by a 30-day potentially avoidable readmission or death [34]. We also performed the test for equality of C-statistics (STATA; roccomp test) to compare C-statistics between SDH-augmented models and the model without SDH. We also calculated the Brier score (< 0.25 is considered useful), which quantifies how close predictions are to the actual outcome (overall performance) [34]. To evaluate calibration, we performed the Hosmer-Lemeshow goodness of fit test, which compares the differences between the predicted and observed outcomes for each decile of risk and tests the statistical significance of the difference. A *p* value ≥ 0.05 for the significance test means that the predicted and observed outcomes are consistent, implying goodness of fit for the predictive model [21, 22]. Finally, we calculated the continuous net reclassification improvement (NRI) for readmitted/deceased patients, non-readmitted/deceased patients, and all patients. NRI examines the net percentage of persons with (without) the event of interest correctly assigned a higher (lower) predicted risk by adding new predictors [46, 47]. For readmitted/deceased patients, the NRI equals to (number of patients with increased predicted risk–number of patients with decreased predicted risk)/number of readmitted/deceased patients. For non-readmitted/deceased patients, the NRI equals to (number of patients with decreased predicted risk–number of patients with increased predicted risk)/number of non-readmitted/deceased patients [46].

All analyses were completed using Stata/MP version 14 (StataCorp).

## Results

### Patient and readmission characteristics

Our study included 19,941 index admissions from 12,537 unique patients during the study period (Table 2). Among all admissions, 3,019 (15.1%) were followed by a potentially avoidable readmission or death 30-day after discharge. Of all patients, the average age was 62.4 and approximately 48.3% were under 65. More than half of patients were female. Approximately 35.3% of patients were white, 47.4% were non-white, and 17.2% were unknown. More than half were non-Hispanic. 76.1% of all patients reported speaking English as a primary language, and 36.2% were married or partnered. Approximately 85.7% of patients were covered by Medicare, Medicaid, or other public insurance programs. More than 83% of patients were admitted through emergency department and 18.6% of all patients had BMI over 30. The average Charlson comorbidity score was 4.0, and the average HOSPITAL score was 2.7.

**Table 2:** Patient characteristics, by readmission or death status.

Patient Characteristics	All (N = 19,941)	With 30-day readmission or death (N = 3,019)	Without 30-day readmission or death (N = 16,922)	P value
**Individual characteristics**				
**Age, mean (SD)**	62.4 (17.0)	63.5 (16.5)	62.2 (17.1)	<0.001
**Age (%)**				
18–34	9.1	7.6	9.4	0.004
35–54	20.6	19.4	20.9	
55–64	18.6	18.9	18.6	
65–74	21.8	22.7	21.7	
75–84	24.6	25.4	24.5	
> = 85	5.2	6.0	5.0	
**Gender (%)**				
Female	51.3	51.7	51.2	0.57
Male	48.7	48.2	48.8	
**Race (%)**				
White	35.3	34.7	35.5	<0.001
Black	18.0	19.5	17.7	
Asian	10.0	8.2	10.4	
American Indian or Alaska Native	0.1	0.0	0.2	
Native Hawaiian or other pacific islander	0.4	0.5	0.4	
Other	18.9	16.8	19.3	
Unknown	17.2	20.3	16.6	
**Ethnicity (%)**				
Non-Hispanic	51.6	51.7	51.6	0.01
Hispanic	15.2	16.8	14.9	
Unknown/declined/other	33.2	31.5	33.5	
**Primary language (%)**				
English speaking	76.1	78.8	75.6	<0.001
Non-English speaking	23.9	21.2	24.4	
**Marital status (%)**				
Married or partnered	36.2	37.2	36.0	0.24
Single/divorced/widowed	63.8	62.8	64.0	
**Insurance (%)**				
Commercial	14.3	8.7	15.3	<0.001
Medicare	28.0	29.5	28.7	
Medicaid	24.2	22.8	24.4	
Dual-eligible	28.8	33.9	27.0	
Other public	4.7	5.2	4.6	
**Admitting Source**				
Emergency department	83.9	85.9	83.5	0.001
Other	16.1	14.1	16.4	
**BMI (%)**				
>30	18.6	20.3	18.6	0.03
**Charlson comorbidity score**	4.0	5.0	3.9	<0.001
**HOSPITAL score, mean**	2.7	3.6	2.6	<0.001
Low risk (0–4)	87.5	74.5	89.8	<0.001
High risk (> = 5)	12.5	25.5	10.2	
**Community characteristics**				
Median income ($)	103,860	104,402	103,763	0.67
Unemployment rate (%)	7.84	7.93	7.83	0.31
% with high school or high school-equivalent diploma	17.82	18.13	17.77	0.10
% foreign born	31.90	30.98	32.06	<0.001
% without insurance	11.17	11.27	11.15	0.43
% dual-eligible	1.37	1.40	1.36	0.17
Felony rate	19.26	18.44	19.40	0.22
Walkability score	2.80	2.59	2.84	<0.001
Gini income inequality coefficient	0.51	0.51	0.51	0.49
The composite score reflecting household composition and disability	0.39	0.40	0.39	0.03
The composite score for minority status and language	0.65	0.64	0.65	0.12

P-values indicate the statistical significance of the difference in patient characteristics between patients with 30-day readmission or death and those without 30-day readmission or death.

Compared to patients without potentially avoidable 30-day readmission or death, those with potentially avoidable 30-day readmission or death were older (63.5 versus 62.2), more likely to be black (19.5% versus 17.7%) and Hispanic (16.8% versus 14.9%), more likely to speak English as a primary language (78.8% versus 75.6%), more likely to be dually enrolled in Medicare and Medicaid (33.9% versus 27.0%), more likely to be admitted from the ED (85.9% versus 83.5%), and more likely to have a BMI over 30 (20.3% versus 18.6%). Patients with avoidable 30-day readmission or death also had higher Charlson comorbidity scores (5.0 versus 3.9), and higher HOSPITAL scores (3.6 versus 2.6). Patients with avoidable 30-day readmission or death also had higher proportion of foreign-born residents, lower walkability score, and poorer conditions in household composition and disability in their neighborhood.

### Risk prediction for potentially avoidable 30-day readmission or death, all patients

The Simplified HOSPITAL score performed similarly on our data set as compared to the original paper [33], with a C-statistic of 0.66 (95% CI: 0.64, 0.68), Brier score of 0.12, and good calibration (Hosmer-Lemeshow goodness-of-fit P = 0.08). Adding individual-level SDH did not produce a statistically significant improvement in the C-statistic, had no effect on the Brier score, and led to better calibration (C-statistic: 0.67; Brier score: 0.12; Hosmer-Lemeshow goodness-of-fit P = 0.15). Adding community-level SDH yielded similar results (C-statistic 0.67; Brier score: 0.12; Hosmer-Lemeshow goodness-of-fit P = 0.20) (Table 3).

**Table 3:** Performance of the predictive model for 30-day readmission or death.

Cohort	Model	C-statistic And 95% CI	Brier score	Hosmer-Lemeshow goodness-of-fit test
**All patients**	Model 1: Simplified HOSPITAL score	0.66 (0.64, 0.68)	0.12	0.08
	Model 2: Simplified HOSPITAL score + individual SDH	0.67 (0.65, 0.69)	0.12	0.15
	Model 3: Simplified HOSPITAL score + census tract SDH	0.67 (0.65, 0.69)	0.12	0.20
	Model 4: Simplified HOSPITAL score + individual SDH + census tract SDH	0.67 (0.65, 0.69)	0.12	0.25
**Medicaid**	Model 1: Simplified HOSPITAL score	0.70 (0.65, 0.75)	0.09	<0.001
	Model 2: Simplified HOSPITAL score + individual SDH	0.71 (0.66, 0.76)	0.09	0.006
	Model 3: Simplified HOSPITAL score + census tract SDH	0.72 (0.67, 0.77)	0.09	0.002
	Model 4: Simplified HOSPITAL score + individual SDH + census tract SDH	0.73 * (0.68, 0.78)	0.09	0.01
**65 and older**	Model 1: Simplified HOSPITAL score	0.66 (0.63, 0.69)	0.11	<0.001
	Model 2: Simplified HOSPITAL score + individual SDH	0.68 * (0.65, 0.71)	0.11	<0.001
	Model 3: Simplified HOSPITAL score + census tract SDH	0.67 (0.63, 0.70)	0.11	<0.001
	Model 4: Simplified HOSPITAL score + individual SDH + census tract SDH	0.68 * (0.65, 0.71)	0.11	<0.001
**Obese**	Model 1: Simplified HOSPITAL score	0.70 (0.63, 0.77)	0.10	<0.001
	Model 2: Simplified HOSPITAL score + individual SDH	0.71 (0.65, 0.77)	0.10	<0.001
	Model 3: Simplified HOSPITAL score + census tract SDH	0.72 * (0.66, 0.79)	0.10	0.001
	Model 4: Simplified HOSPITAL score + individual SDH + census tract SDH	0.73 * (0.67, 0.79)	0.10	0.001

CI: confidence interval. SDH: social determinants of health; individual SDH include sex, race (i.e., White, African American, Asian, American Indian/Alaska Nation, Native Hawaiian/Pacific Islander, other, and unknown), ethnicity (i.e., non-Hispanic, Hispanic, and unknown/declined/other), primary language (i.e., English vs. other), marital status (i.e., partnered vs. single), and insurance type (i.e., commercial, Medicare, Medicaid, dual-eligible for Medicare and Medicaid, and other public insurance). Community-level SDH include socioeconomic status (i.e., median income, unemployment rate, % with high school or high school-equivalent diploma, % foreign born, % without insurance, and % dual-eligible); felony rate, walkability score, Gini income inequality coefficient, a composite score reflecting household composition and disability, and a composite score for minority status and language. Asterisk indicates the significance of the difference in c-statistics between model 1 and model 2–4. * P<0.05, ** P<0.01, *** P<0.001.

### Risk prediction for potentially avoidable 30-day readmission, subgroup analyses

In accordance with our second hypothesis, we examined performance of the predictive model in the three vulnerable subgroups (Table 3).

Within vulnerable subgroups, the Simplified HOSPITAL score produced better discrimination (C-statistic: 0.66–0.70) and fairly similar accuracy (Brier score: 0.09–0.11) as compared to the overall population. Calibration was poor for all subgroups (Hosmer-Lemeshow goodness-of-fit P < 0.05). Adding individual-level SDH made statistically significant improvements in the C-statistic for patients 65 or older and calibration for Medicaid patients without affecting the Brier score. Adding community-level SDH produced greater improvements in the C-statistic among obese patients (C-statistic: 0.72; 95% CI [0.66, 0.79]).

Incorporating both individual and community-level SDH significantly improved discrimination among all subgroups but had little impact on accuracy or calibration.

Collinearity diagnostics indicated sufficiently low multicollinearity in all models. The highest VIF value is 4.2, which is lower than the widely used threshold of 10 to indicate multi-collinearity [48].

For all patients and patients in each subgroup, the event NRI (for readmission or death) was positive when adding individual SDH, community SDH, or both (Table 4), indicating that a higher proportion of patients got assigned a higher predicted risk correctly when adding SDH. The NRIs were especially higher for patients in three subgroups than overall patients. The nonevent NRI (for no readmission or death) was negative among some groups. The overall NRI (the sum of event and nonevent NRI) still improved after adding SDH.

**Table 4:** Net reclassification improvement after adding SDH predictors.

Cohort	Model	Event NRI	Nonevent NRI
**All patients**	Model 1: Simplified HOSPITAL score	–	–
	Model 2: Simplified HOSPITAL score + individual SDH	15.5%	-1.3%
	Model 3: Simplified HOSPITAL score + census tract SDH	10.6%	-6.7%
	Model 4: Simplified HOSPITAL score + individual SDH + census tract SDH	14.6%	2.2%
**Medicaid**	Model 1: Simplified HOSPITAL score	–	–
	Model 2: Simplified HOSPITAL score + individual SDH	2.8%	13.5%
	Model 3: Simplified HOSPITAL score + census tract SDH	24.8%	2.2%
	Model 4: Simplified HOSPITAL score + individual SDH + census tract SDH	13.8%	21.2%
**65 and older**	Model 1: Simplified HOSPITAL score	–	–
	Model 2: Simplified HOSPITAL score + individual SDH	28.4%	-12.4%
	Model 3: Simplified HOSPITAL score + census tract SDH	11.8%	-4.0%
	Model 4: Simplified HOSPITAL score + individual SDH + census tract SDH	21.2%	-5.4%
**Obese**	Model 1: Simplified HOSPITAL score	–	–
	Model 2: Simplified HOSPITAL score + individual SDH	23.5%	-14.6%
	Model 3: Simplified HOSPITAL score + census tract SDH	41.2%	-3.7%
	Model 4: Simplified HOSPITAL score + individual SDH + census tract SDH	32.4%	-3.6%

NRI: net reclassification improvement; individual SDH include sex, race (i.e., White, African American, Asian, American Indian/Alaska Nation, Native Hawaiian/Pacific Islander, other, and unknown), ethnicity (i.e., non-Hispanic, Hispanic, and unknown/declined/other), primary language (i.e., English vs. other), marital status (i.e., partnered vs. single), and insurance type (i.e., commercial, Medicare, Medicaid, dual-eligible for Medicare and Medicaid, and other public insurance). Community-level SDH include socioeconomic status (i.e., median income, unemployment rate, % with high school or high school-equivalent diploma, % foreign born, % without insurance, and % dual-eligible); felony rate, walkability score, Gini income inequality coefficient, a composite score reflecting household composition and disability, and a composite score for minority status and language.

## Discussion

Despite evidence that SDH are significantly associated with health outcomes, we found that incorporating individual- or community-level SDH did not meaningfully improve the prediction of potentially avoidable 30-day readmission for a general patient population. However, adding individual- or community-level SDH improved model performance among patient subgroups who may not be able to compensate for social risk factors, namely Medicaid patients, patients who are 65 or older, and obese patients.

Our results are consistent with previous literature, which found that adding SDH information did not significantly improve the prediction of some health-related outcomes [49, 50]. It is possible that SDH are correlated with the original predictors representing patient comorbidity and health status linked to the index admission, and therefore added little to the prediction of 30-day readmission. Previous literature indicated that SDH are associated with increased risk for various medical and behavioral conditions [51]. Another explanation could be that some individual-level SDH variables not documented in the EHR, such as income, education, and occupation, could have stronger predictive power.

It is also plausible that readmission is primarily determined by clinical factors captured in the HOSPITAL score, or by other process factors not captured in our model, such as care delivery processes and provider-level variables [16, 52]. Existing risk prediction tools usually model readmission based on patient characteristics, with the assumption that patient demographics, socioeconomics, and comorbidities are key determinants of readmission [17, 20]. However, previous literature has also indicated that care delivery characteristics, such as discharge planning and care coordination, are also strongly associated with hospital readmission [52]. Incorporating these factors may improve the risk prediction of readmission.

We found adding SDH improved readmission risk prediction for vulnerable subgroups, including Medicaid patients, patients who are 65 or older, and obese patients. Medicaid patients with low income are disproportionately likely to have health-related unmet social needs, such as food and housing [53]. In addition, these patients may be less likely to compensate for disadvantaged neighborhood social conditions (e.g., poor transportation conditions or lack of access to high-quality groceries) as compared to other patients with higher socioeconomic status in similar neighborhoods [54]. Our second vulnerable subgroup, patients who are 65 or older, are often affected by multiple chronic conditions, cognitive disability, and social isolation. Adverse neighborhood social conditions may exacerbate the effects of these conditions, leaving them at higher risk for readmission. Finally, obese patients have been identified as having a higher risk for social vulnerability and having higher readmission rates after surgery. Obesity, coupled with other social vulnerabilities, such as advanced age, disability, or minority background, can result in a synergistic effect, amplifying the combined impact of these factors [55–58]. This may render patients with obesity particularly vulnerable to the effects of social risk factors.

This study has several limitations. First, we used EHR data from a single academic medical center. Patients might have been readmitted to hospitals other than the index hospital, artificially deflating readmission counts. This would create particularly strong biases if patients with disadvantaged social conditions are more likely to be readmitted to other hospitals, which, if true, would be likely to bias our analyses toward the null. Second, this study was based on a group of patients from New York City, and our results may not be generalizable to other populations. For example, a key transportation variable in New York City is proximity to public transportation. In other regions of the US, access to a car may be a more important variable. Third, some important individual-level SDH, such as income, education, and occupation were not available in this study. Adding these SDH may have a different impact on model performance. Fourth, SDH and clinical factors may be associated with readmissions in interactive ways. Using machine learning-based methods may be able to identify important interaction terms. Fifth, capture of post-discharge mortality is poor in EHR data. Further research may focus on supplementing the data set with other sources, including both insurance claims data and data from other NYC healthcare system, such as organizations participating in centralized clinical data research networks [59], to address these limitations.

## Conclusion

We examined the value of SDH in predicting potentially avoidable 30-day readmission and we found SDH did not improve risk prediction for the overall patient population. However, SDH improved the performance of models for three vulnerable patient populations, namely, Medicaid patients, obese patients, and patients 65 and older. Future studies may examine more SDH that are related to readmission and develop the prediction model based on other feature selection methods.

## Supporting information

S1 Appendix (DOCX)
