# Supplementary material for: Assessing the impact of social determinants of health on predictive models for potentially avoidable 30-day readmission or death
Source: PLoS One. 2020 Jun 25;15(6):e0235064. doi: 10.1371/journal.pone.0235064 (PMC7316307; doi:10.1371/journal.pone.0235064)
Supplement: S1 Appendix — (DOCX) [file pone.0235064.s001.docx]

**Assessing the Impact of Social Determinants of Health on Predictive Models for Potentially Avoidable 30-Day Readmission or Death**

Supporting Table S1 All Individual and Community Social Determinants of Health Variables Considered in the Study

| Social Determinants of Health Variables | Values | Source |
| --- | --- | --- |
| Individual level |  |  |
| Sex | Male, female | EHR |
| Race | White, African American, Asian, American Indian/Alaska Nation, Native Hawaiian/Pacific Islander, other, and unknown | EHR |
| Ethnicity | Non-Hispanic, Hispanic, and unknown/declined/other | EHR |
| Primary language | English, other | EHR |
| Marital status | Partnered, single | EHR |
| Insurance | Commercial, Medicare, Medicaid, dual-eligible for Medicare and Medicaid, and other public insurance | EHR |
| Census tract level |  |  |
| Median income | Median income in a census tract | ACS |
| % below federal poverty level | % of household below federal poverty level in a census tract | ACS |
| % Medicaid | % of residents with Medicaid coverage in a census tract |  |
| Unemployment rate | Unemployment rate in a census tract | ACS |
| % with high school or high school-equivalent diploma | % of residents 25 years and over with high school or high school-equivalent diploma in a census tract | ACS |
| % foreign born | % if foreign-born population in a census tract | ACS |
| % without insurance | % of residents without insurance coverage in a census tract | ACS |
| % dual-eligible | % of residents who are dually eligible for both Medicare and Medicaid | ACS |
| Felony rate | Number of felonies per 1,000 people in the census tract | NYC Open Data |
| Violation rate | Number of violations per 1,000 people in the census tract | NYC Open Data |
| Misdemeanor rate | Number of misdemeanors per 1,000 people in the census tract | NYC Open Data |
| Walkability score | Neighborhood walkability scale | FACETS |
| Bus stop | Number of bus stops in a census tract | NYC Open Data |
| Subway station | Number of subway stations in a census tract | NYC Open Data |
| Gini income inequality coefficient | Gini income inequality coefficient in a census tract | ACS |
| The composite score reflecting household composition and disability | The score of the household composition & disability theme of the Social Vulnerability Index in a census tract | CDC |
| The composite score for minority status and language | The score of the minority status & language theme of the Social Vulnerability Index in a census tract | CDC |
| The composite score for socioeconomic | The score of the **s**ocioeconomic theme of the Social Vulnerability Index in a census tract | CDC |
| The composite score for housing type & transportation | The score of the housing type & transportation theme of the Social Vulnerability Index in a census tract | CDC |
| Respiratory hazard index | A score indicating respiratory risk in the air | CDC |
| Tree count | Number of trees in the community | NYC Open Data |

*Notes: EHR: electronic health record; ACS: American Community Survey; CDC: Centers for Disease Control and Prevention. To allow for nonlinear associations between community SDH and readmission, we incorporated census-tract level SDH variables as community deciles in the analysis.*

Supporting Table S2 Sources of Social Determinants of Health Variables Used in the Study

| Social Determinants of Health Variables | Values | Source |
| --- | --- | --- |
| Individual level |  |  |
| Sex | Male, female | EHR |
| Race | White, African American, Asian, American Indian/Alaska Nation, Native Hawaiian/Pacific Islander, other, and unknown | EHR |
| Ethnicity | Non-Hispanic, Hispanic, and unknown/declined/other | EHR |
| Primary language | English, other | EHR |
| Marital status | Partnered, single | EHR |
| Insurance | Commercial, Medicare, Medicaid, dual-eligible for Medicare and Medicaid, and other public insurance | EHR |
| Census tract level |  |  |
| Median income | Median income in a census tract | ACS |
| Unemployment rate | Unemployment rate in a census tract | ACS |
| % with high school or high school-equivalent diploma | % of residents 25 years and over with high school or high school-equivalent diploma in a census tract | ACS |
| % foreign born | % if foreign-born population in a census tract | ACS |
| % without insurance | % of residents without insurance coverage in a census tract | ACS |
| % dual-eligible | % of residents who are dually eligible for both Medicare and Medicaid | ACS |
| Felony rate | Number of felonies per 1,000 people in the census tract | NYC Open Data |
| Walkability score | Neighborhood walkability scale | FACETS |
| Gini income inequality coefficient | Gini income inequality coefficient in a census tract | ACS |
| The composite score reflecting household composition and disability | The score of the household composition & disability theme of the Social Vulnerability Index in a census tract | CDC |
| The composite score for minority status and language | The score of the minority status & language theme of the Social Vulnerability Index in a census tract | CDC |

*Notes: EHR: electronic health record; ACS: American Community Survey; CDC: Centers for Disease Control and Prevention. To allow for nonlinear associations between community SDH and readmission, we incorporated census-tract level SDH variables as community deciles in the analysis.*

Supporting Table S3 Logistic Regression Results after Adding Both Individual and Community SDH in the Model

|  | All patients | Medicaid | 65 or older | Obese |
| --- | --- | --- | --- | --- |
|  | Odds Ratio | Odds Ratio | Odds Ratio | Odds Ratio |
| Hospital score | 1.37 *** | 1.39 *** | 1.31 *** | 1.36 *** |
| Individual SDH |  |  |  |  |
| *Gender (ref. female)* |  |  |  |  |
| Male | 0.98 | 1.12 | 0.91 | 1.25 |
| *Race (ref. White)* |  |  |  |  |
| Black | 0.98 | 0.93 | 079 | 1.08 |
| Asian | 0.78 | 0.89 | 0.89 | 0.61 |
| American Indian or Alaska Native | 0.29 | -- | 0.73 | -- |
| Native Hawaiian or other pacific islander | 1.55 | 0.45 | 1.80 | -- |
| Other | 1.14 | 1.17 | 1.21 | 1.08 |
| Unknown | 0.88 | 0.78 | 1.03 | 0.91 |
| *Ethnicity (ref. non-Hispanic)* |  |  |  |  |
| Hispanic | 0.97 | 1.02 | 1.01 | 1.23 |
| Unknown/declined/ other | 0.91 | 0.97 | 0.97 | 1.05 |
| *Primary language (ref. English)* |  |  |  |  |
| Non-English speaking | 0.80 ** | 0.87 | 0.73 ** | 1.12 |
| *Marital status (ref. married)* |  |  |  |  |
| Single / divorced / widowed | 0.86 * | 0.86 | 0.86 * | 1.02 |
| *Insurance (ref. commercial)* |  |  |  |  |
| Medicare | 1.43 ** | -- | 1.84 | 1.69 * |
| Medicaid | 1.44 ** | -- | 1.32 | 1.52 |
| Dual-eligible | 1.85 *** | -- | 2.27 | 2.04 ** |
| Other public | 1.66 ** | -- | 2.42 | 1.92 * |
| Community characteristics |  |  |  |  |
| Median income ($) | 1.00 | 1.00 | 1.00 | 1.00 |
| Unemployment rate (%) | 1.00 | 0.99 | 1.00 | 0.99 |
| % with high school or high school-equivalent diploma | 1.00 | 1.01 | 0.99 | 1.00 |
| % foreign born | 0.99 | 0.99 | 0.99 | 0.99 |
| % dual-eligible | 1.02 | 1.00 | 1.03 | 1.03 |
| % without insurance | 1.00 | 0.99 | 1.01 | 1.01 |
| Felony rate | 0.99 | 0.99 | 0.99 | 1.00 |
| Walkability score | 1.01 | 0.98 | 1.01 | 1.00 |
| Gini income inequality coefficient | 0.69 | 0.95 | 0.49 | 0.25 |
| Household composition and disability score | 1.04 | 1.04 | 1.27 | 1.27 |
| Minority status and language score | 0.83 | 0.89 | 0.65 | 0.77 |

*Notes: * P<0.05, ** P<0.01, *** P<0.001.*

**References:**

1. United States Census Bureau [Internet]. Suitland: American Community Survey; 2020 [cited 2020 01/31]. Available from: https://www.census.gov/programs-surveys/acs

2. Centers for Disease Control and Prevention [Internet]. Atlanta: CDC's Social Vulnerability Index (SVI); 2018 [cited 2019 05/01]. Available from: <https://svi.cdc.gov/>.

3. Cantor MN, Chandras R, Pulgarin C. FACETS: using open data to measure community social determinants of health. J Am Med Inform Assoc. 2018;25(4):419-22. Epub 2017/11/02. doi: 10.1093/jamia/ocx117. PubMed PMID: 29092049; PubMed Central PMCID: PMCPMC5885799.

4. City of New York [Internet]. New York: New York City Open Data; 2017 [cited 2019 12/2]. Available from: <https://opendata.cityofnewyork.us/>.
